# Editorial: The application of artificial intelligence in diagnosis, treatment and prognosis in urologic oncology

**DOI:** 10.3389/fonc.2022.1118442

**Published:** 2022-12-21

**Authors:** Xue-hua Zhu, Chin-Lee Wu, Xiong-bing Zu, Jian Lu

**Affiliations:** ^1^ Department of Urology, Peking University Third Hospital, Beijing, China; ^2^ Department of Urology, Massachusetts General Hospital, Harvard Medical School, Boston, MA, United States; ^3^ Department of Pathology, Massachusetts General Hospital, Harvard Medical School, Boston, MA, United States; ^4^ Department of Urology, National Clinical Research Center for Geriatric Disorders, Xiangya Hospital, Central South University, Changsha, China

**Keywords:** artificial intelligence (AI), machine learning, urological oncology, radiomics, clinical transformation

Artificial intelligence (AI) is reshaping and improving traditional decision-making patterns. Although the further integration of AI into clinical practice will take time, research in this field has been producing promising results. AI has the capability to aid in several aspects of disease management. In the specialty of oncology, for example, AI can assist in disease diagnosis, classification, treatment optimization, therapeutic response evaluation, prognosis prediction, and follow-up scheduling. The greatest strength of AI is its capability to extract and interpret information from a large amount of medical data, which is included in images, pathological films, and the electronic reporting system. AI can process and analyze clinical data, but the technology cannot be fully utilized by clinicians in an advanced, efficient, and intelligent manner in order to aid in clinical decision-making.

This edition of Frontiers in Oncology seeks to assist physicians in completely comprehending and accepting AI’s critical role and great potential in solving actual clinical problems, as well as to accelerate the clinical translation of relevant scientific findings. This issue consists of 10 manuscripts (including 8 original papers and 2 reviews) on the application of AI to urologic oncology and benign disease. Liu et al. develop a diagnostic tool utilizing deep learning systems (DLSs) to detect malignant cells in urine cytopathological images and then predict the histopathological results. The DLSs model achieves good predictive performance in both the internal set and extra set with an area under the curve (AUC) of 0.90 and 0.93, respectively. The model is expected to enhance the efficacy of urine biopsies and aid in the early detection and risk classification of patients with UC. The prediction model based on a large sample database can estimate the prognosis of patients more precisely and aid in the establishment of individualized treatment plans and follow-up care. Zhang et al. created a nomogram based on the SEER database to predict cancer-specific survival (CSS) and overall survival (OS) among the elderly PCa group. This model offers precise predictions for CSS and OS in both the training and validation sets. Deng et al. develop five different machine learning (ML) models to predict the presence of prostate cancer (PCa) in patients with PSA ≤ 20 ng/mL. The random forest (RF) model performs the best among five models, with an AUC of 0.871 in the training cohort and 0.78 in the validation cohort. The research conducted by Chen et al. also focuses on the PCa prediction. Based on traditional clinical variables, five different ML models are developed and compared in terms of their predictive performance. In the test dataset, the multivariate univariate logistic regression (LR) model exhibited the best discrimination (AUC=0.918). The predictive model built by Zhang and Chen can be utilized for preliminary screening of individuals with suspected PCa, potentially reducing the number of unnecessary biopsies and missed PCa diagnoses.

Radiomics is the integration of AI with image data. The extraction and interpretation of image data expands the clinician’s insight beyond the visible conventional information like lesion size, location, and shape. The acquisition of the region of interest (ROI) is a crucial step in the radiomics workflow. Manually drawing ROI is the most common method, but it is laborious and time-consuming. Chen et al. present a radiomics model based on auto-segmentation of ultrasound images to detect diabetic kidney disease (DKD) patients in three centers. DeepLabV3+ network, a DL-based anatomical-level segmentation system, demonstrates a good segmentation ability with mean pixel accuracy in the three centers of 0.890 ± 0.004, 0.870 ± 0.002 and 0.893 ± 0.007, respectively. The radiomics model delivers a fair ability for DKD recognition (AUC: 0.674 ± 0.074) and a good discriminative capability for DKD stage (AUC: 0.803 ± 0.037). Zhu et al. establish a cascaded DL model to automatically segment the whole prostate gland, the anatomic zones, and the csPCa region step by step based on biparametric MRI. The model employing cascaded convolutional neural networks (CNNs) could automatically detect and segment the suspicious csPCa lesions with excellent performance (sensitivity=95.6%, specificity=91.5%, accuracy= 92%) on MR images without any human intervention.

Ultrasound is another important imaging method for PCa diagnosis. Wang et al. propose a ML model based on 14 features extracted from transrectal ultrasound video clips of the whole prostate gland. The support vector machine (SVM) and random forest (RF) algorithms were used to establish radiomics models based on those features. The SVM model exceeds radiologist’s diagnostic ability based on MRI. Imaging provides information regarding the heterogeneity and microenvironment of a tumor. Yang et al. develop and externally validate a Transformer-based DL algorithm with CT images to predict the Fuhrman nuclear grade of clear cell renal cell carcinoma (ccRCC). TransResNet, a framework network integrating CNNs, self-attention mechanisms and nonlinear classifier, outperforms conventional DL algorithms for ccRCC grade prediction with an AUC of 91.2%. This work presents a non-invasive approach for predicting the pathological grade of ccRCC, hence avoiding the complications associated with puncture biopsy.

Zhang et al. investigated the diagnostic performance of CT-radiomics in adrenal mass using a systematic review and meta-analysis. The quantitative analysis of nine studies shows that CT-based radiomics may help identify malignant adrenal tumors from benign ones with pooled sensitivity, specificity, and AUC of 0.80, 0.83, and 0.88, respectively. A mini-review by Huang et al. contains 12 articles concerning the use of radiomics in the prediction of muscle-invasive bladder cancer (BC). Radiomics utilizing CT or MRI images has the potential to detect muscle invasion.

The powerful combination of AI and medical data has yielded significant achievements in the last twenty years. Some achievements have been clinically transformed and accepted by clinicians. Studies with prospective design is required to expedite the clinical translation of AI-related findings. AI researchers should not only concentrate on the optimization of algorithms and the performance of models but also explore when and how to actualize the clinical utility of AI’s achievements.

## Author contributions

All authors listed have made a substantial, direct, and intellectual contribution to the work and approved it for publication.

